# Insecticidal Potential of *Aniba canelilla* (H.B.K.) Mez Essential Oil Against *Aedes aegypti*: Larvicidal and Adulticidal Activities, Mechanism of Action, and Formulation Development

**DOI:** 10.3390/plants14213348

**Published:** 2025-10-31

**Authors:** Jefferson D. da Cruz, Maíra M. H. Almeida, Maria Athana M. Silva, Jefferson R. A. Silva, Fernando A. Genta, Ana Claudia F. Amaral

**Affiliations:** 1 Laboratório de Produtos Naturais e Derivados, Departamento de Produtos Naturais, Farmanguinhos/Fiocruz, Manguinhos, Rio de Janeiro 21041-250, RJ, Brazil; jefferson_dacruz@hotmail.com (J.D.d.C.); maira-haddad@hotmail.com (M.M.H.A.); maria.mpalantinos@fiocruz.br (M.A.M.S.); 2 Programa de Pós-Graduação Acadêmica em Pesquisa Translacional em Fármacos e Medicamentos, Farmanguinhos/Fiocruz, Manguinhos, Rio de Janeiro 21041-250, RJ, Brazil; 3 Laboratório de Cromatografia, Departamento de Química, Instituto de Ciências Exatas, Universidade Federal do Amazonas, Manaus 69077-000, AM, Brazil; jrocha_01@yahoo.com.br; 4 Laboratório de Bioquímica e Fisiologia de Insetos, Instituto Nacional de Endemias Rurais (INERu), IOC/Fiocruz, Rio de Janeiro 21040-360, RJ, Brazil; 5 Laboratório de Bioquímica e Fisiologia de Insetos, Instituto Oswaldo Cruz, IOC/Fiocruz, Rio de Janeiro 21040-360, RJ, Brazil

**Keywords:** Lauraceae, mosquito, insecticidal activity, arbovirose, enzyme inhibition, nanoformulation

## Abstract

Control of *Aedes aegypti*, the primary vector of arboviruses such as dengue, Zika, and chikungunya, is increasingly difficult due to resistance to synthetic insecticides and environmental concerns. Plant essential oils offer sustainable alternatives with multi-target modes of action and rapid biodegradation. This study evaluated the insecticidal potential of the essential oil of *Aniba canelilla* (EOANIB), its major constituent 1-Nitro-2-phenylethane (NFTANE), and the derivative 1-Nitro-2-phenylethene (NFTENE) against larvae and adults of *A. aegypti*. Acetylcholinesterase (AChE) inhibition was quantified using enzymes from *Electrophorus electricus*, *Aedes aegypti* and *Drosophila melanogaster*. Pluronic^®^ F127 (5% *w*/*v*) nanoformulations loaded with EOANIB, NFTANE, or NFTENE at 1.5% or 0.34% (*w*/*v*) improved efficacy and stability. Formulations remained stable for 120 to 190 days at 25 to 60 °C. Larvicidal assay at 24 h yielded LC_50_ values of 86.9 (CI 78.2–94.7) ppm for EOANIB, 84.8 ppm (CI 75.6–92.4) for NFTANE and 10.9 (CI 8.0–14.0) ppm for NFTENE. Against adults, EOANIB achieved an LC_50_ of 33.9 ppm at 1.5 h. Nanoformulation reduced the EOANIB LC_50_ by 22.2% after 24 h and 40.1% after 48 h. Toxicity assays evaluated selectivity with *Artemia salina* (EOANIB LC_50_: 77.2 ppm) and no mortality in *D. melanogaster* at 100 ppm. The convergence of efficacy, formulation-enhanced performance, and demonstrated storage stability positions *Aniba canelilla* as a promising source of bioinsecticide candidates for *Aedes aegypti* control and supports further development of micellar delivery systems for integrated vector management.

## 1. Introduction

Vector-borne diseases transmitted by insects remain major causes of global morbidity and mortality. The World Health Organization (WHO) recognizes 17 groups of neglected tropical diseases (NTDs), of which 14 occur in Brazil [1]. These diseases are frequently linked to poor sanitation, disproportionately affecting low-income populations and imposing irreversible economic and social burdens [2,3].

Mosquitoes rank among the main vectors of NTDs. Each year, the WHO reports approximately one billion cases and millions of deaths from mosquito-borne pathogens [1,4]. Medically significant species belong primarily to two subfamilies: Anophelinae (genus *Anopheles*) and Culicinae (genera *Aedes*, *Culex*, and *Mansonia*), which are widespread across Asia, the Middle East, and the Americas. These vectors transmit dengue, Zika, chikungunya, lymphatic filariasis, malaria, yellow fever, and Japanese encephalitis [4].

Within the Culicinae subfamily, *Aedes aegypti* is the principal urban arbovirus vector, occurring in 128 tropical and temperate countries. This species transmits dengue, Zika and chikungunya, three NTDs with major global health impact. Dengue is the most prevalent arboviral disease worldwide, responsible for an estimated 390 million infections annually [5]. In Brazil, *A. aegypti* has accounted for most chikungunya outbreaks recorded since 2000 [6] and for congenital malformations linked to Zika virus infection [7].

Recent epidemiological trends underscore an alarming expansion of these arboviruses. In 2024, more than 11 million dengue cases were reported in the Americas, an increase of 255% over 2023 with 6600 confirmed deaths. Brazil accounted for 82.4%, and all four dengue serotypes (DENV-1 to DENV-4) circulate concurrently in the country [8]. The geographic spread of *Aedes* into previously non-endemic areas represents an additional global threat. In Europe, 122 autochthonous dengue cases were reported in 2023 (Italy, France, and Spain), marking a 184% increase compared to 2022, with Italy alone confirming 82 cases [9,10]. In the Americas, 271,006 chikungunya and 36,340 Zika cases were recorded in 2022 [11].

In the absence of widely available vaccines for chikungunya and Zika, and with inadequate dengue vaccine coverage, chemical control remains the predominant strategy owing to its low cost and rapid action. Nonetheless, urgent development of more effective and environmentally sustainable approaches is necessary [3,12].

Conventional chemical control is subject to serious long-term limitations. Synthetic insecticides exhibit environmental toxicity and harm non-target organisms, including pollinators essential to ecosystem services. In humans, organophosphates cause acute toxicity via acetylcholinesterase inhibition, leading to reduced concentration and memory, depression, delayed reaction time, and insomnia [13,14]. Widespread resistance to all major insecticide classes currently used against *Aedes aegypti* and *Aedes albopictus* has been documented across the Americas, Africa, and Asia [15], highlighting the need for alternative chemistries. Essential oils represent a viable option as biodegradable mixtures with selective activity and favorable safety profiles, acting through multiple neurophysiological pathways, including neurotoxic, cytotoxic, and mutagenic effects on target insects [14,16].

Against this background, the Amazonian tree *Aniba canelilla* (H.B.K.) Mez (Lauraceae), traditionally used as a culinary spice, fragrance source, and medicinal ingredient [17,18], is a relevant candidate for investigation. The essential oil of *A. canelilla* exhibits antioxidant, anti-inflammatory, anesthetic, and acetylcholinesterase-inhibitory activities [19,20,21]. The major constituent of the oil, 1-Nitro-2-phenylethane (NFTANE), shows insecticidal activity, partly via acetylcholinesterase inhibition [20,21], supporting further evaluation of entomotoxic potential.

**Figure 1:**
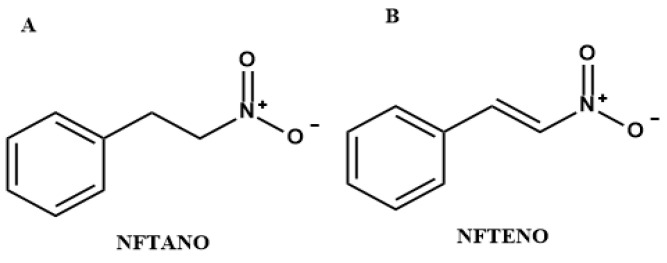
Chemical structure of 1-nitro-2-phenylethane-PubChem CID 80208 (**A**) and 1-phenyl-2-nitroethene-PubChem CID 5284459 (**B**).

The 1-Nitro-2-phenylethene NFTENE, a synthetic derivative structurally related to the natural compound NFTANE, contains a C1–C2 double bond (Figure 1) that restricts molecular rotation. This feature has been associated with enhanced biological activity in drug-like compounds [22,23,24,25,26]. In vivo and in vitro studies have demonstrated that NFTENE exhibits vasodilatory and anti-inflammatory activities [22,27].

The present study evaluated the insecticidal potential of *A. canelilla* essential oil (EOANIB), its major component NFTANE and the synthetic derivative NFTENE against *A. aegypti* larvae and adults. To address physicochemical constraints, namely poor aqueous solubility and high volatility, Pluronic^®^ F127 nanostructured formulations were developed to enhance the bioavailability and performance of these activities [28,29]. Mechanistic evaluation comprised acetylcholinesterase (AChE) inhibition assays using multiple enzyme sources, complemented by selectivity assessments in nontarget organisms. Collectively, the data support sustainable alternatives to conventional insecticides and inform integrated management of the arboviral vector *Aedes aegypti*.

## 2. Results and Discussion

### 2.1. Evaluation of the Essential Oil from Aniba canelilla (EOANIB) and Isolation of 1-Nitro-2-Phenylethane (NFTANE)

Hydrodistillation of 500 g of dried *Aniba canelilla* leaves afforded 4.18 g of essential oil (EOANIB), corresponding to 0.84% (*w*/*w*), which falls within previously reported ranges (0.27–0.80%) [30,31,32]. Gas chromatography–mass spectrometry (GC–MS) of EOANIB (Table 1) identified 1-Nitro-2-phenylethane (NFTANE) as the major constituent (70%), followed by 2-phenylacetaldehyde (4.2%), α-caryophyllene (1.7%), β-caryophyllene (2.2%), and (E)-nerolidol (3.2%), alongside additional trace components. These results are consistent with the high abundance of NFTANE in leaf oil reported in the literature [19,31,32,33]. EOANIB is traditionally rich in 1-Nitro-2-phenylethane (NFTANE), although its relative abundance varies substantially (31.22–95.3%). In contrast, bark-derived essential oils frequently exhibit higher methyleugenol levels (2.9–52.9%), and lower proportions of terpenoids such as linalool, safrole, and limonene, underscoring organ-specific metabolic profiles [23,31,32,33].

Guided by the GC–MS fingerprint of EOANIB, fractionation by Kugelrohr microdistillation was employed to enrich the nitroaromatic fraction and isolate NFTANE (Table 1). The process furnished 0.834 g of NFTANE (57% overall yield) at 99% purity (GC–MS), demonstrating the efficiency of this short-path approach for obtaining a high-purity, volatile constituent.

1-Nitro-2-phenylethane (NFTANE) is an aryl-substituted nitroalkane and a relatively rare plant metabolite, previously reported in the essential oils of *Uvaria chamae* and *Ocotea pretiosa*, as well as in the seeds of *Dennettia tripetala* [35,36,37]. Furthermore, NFTANE shows activity against *Trypanosoma evansi* at 0.5–2.0% [38] and against *Leishmania amazonensis* (IC_50_ of 40 µg/mL), compared with pentamidine (IC_50_ of 4.8 µg/mL). Although approximately eightfold less potent, NFTANE is approximately 2.5-fold less cytotoxic to macrophages [39]. In vivo, NFTANE acts as an arterial vasodilator, significantly lowering blood pressure in Wistar rats at 5–10 mg/kg, with reversible effects and no reported toxicity [40]. Additional pharmacological activities include anxiolytic, anticonvulsant, and antinociceptive effects [41]. GC-MS analysis of the commercial NFTENE sample revealed 88% NFTENE, with benzaldehyde accounting for the remaining 12%. Prior studies have reported anti-inflammatory and vasorelaxant effects for NFTENE [22,27].

### 2.2. Evaluation of Polymeric Micelles Loaded with EOANIB, NFTANE and NFTENE

**Figure 2:**
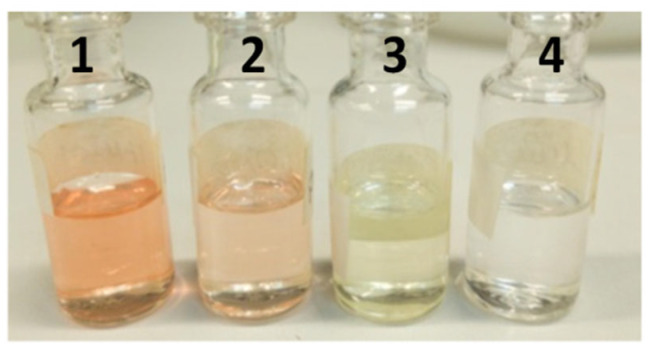
Macroscopic appearance of formulations prepared with Pluronic F-127 and the respective samples. 1: PEOANIB; 2: PNFTANE; 3: PNFTENE; 4: PBlank. Image was recorded one day after preparation and filtration through a 0.45 µm regenerated cellulose (RC) membrane filter.

The primary objective of the formulation was to enhance the chemical stability of the active compounds, increase their biological activity, minimize volatilization losses, and prevent degradation [28,42,43]. Formulations were prepared using the polymeric film hydration method, as described by Santos and coworkers [44]. Polymeric micellar formulations containing the essential oil of *A. canelilla* (PEOANIB), 1-Nitro-2-phenylethane (PNFTANE), and 1-Nitro-2-phenylethene (PNFTENE) displayed high visual stability, as demonstrated in Figure 2.

#### 2.2.1. Zeta Potential (ZP) and Particle Size

The polymeric micelle formulations PEOANIB, PNFTANE, and PNFTENE exhibited zeta potentials of −11.4 mV, −23.1 mV, and −17.8 mV, respectively (Table 2 and Supplementary Figures S1 and S2). The values of −11.4 mV and −17.8 mV fall below the conventional stability range of ±20 to ±30 mV, typically associated with electrostatic stabilization of colloidal systems. However, all formulations displayed adequate physical stability, which is attributed to the non-ionic nature of the Pluronic^®^ F-127 copolymer [45,46]. In such systems, stabilization primarily occurs via non-electrostatic mechanisms, including hydrogen bonding between water molecules and polar groups on the polymer, dipole-induced interactions, and van der Waals forces [47].

Formulations based on non-ionic copolymers generally exhibit zeta potential values close to 0 mV [42]. The incorporation of ionic compounds may increase the ZP without compromising the overall system stability. Evaluation of the cloud point, a visual indicator of instability, revealed no turbidity, corroborating the stability of the formulations. This study evaluated the transmission performance of samples under heat stress up to 65 °C, with no cloud point observed. When the cloud point occurs, the transmittance drops dramatically, a finding that was not observed in the assay. The results demonstrate that up to the limit temperature evaluated (65 °C), the formulation remained stable and acceptable for the proposed study, given that the development temperature of *A. aegypti* larvae and *A. salina* nauplii is around 28–30 °C [48]. The measured ZP values are consistent with previously reported stable non-ionic systems [42,45]. The PEOANIB and PNFTANE formulations exhibited mean particle diameters of 33.4 nm and 32.4 nm, respectively, with polydispersity index (PDI) values of 0.20 and 0.15, and light transmittance values of 87.2% and 86.3%, respectively (Table 3). These PDI values are below the 0.30 threshold, which is considered acceptable for monodisperse systems [49]. The similarity in particle size reflects the comparable physicochemical properties of the essential oil and its major constituent NFTANE (70%). Pluronic F127 based micelles have been reported to range from 3.3 nm (unloaded micelles) [50] to 490 nm when loaded with hydrophobic compounds [42,51].

PNFTENE formulation exhibited a markedly larger mean particle diameter (370 nm, approximately 11-fold increase compared to PEOANIB) and a PDI of 0.13. This enlargement likely reflects the physicochemical profile of the synthetic compound (crystalline solid at ambient temperature), the critical micelle concentration of the polymer, and the influence of ionic strength and co-solvent composition. Consistent with prior reports, adding NaCl or PBS [52] or co-solvents such as DMSO and ethanol [53] can modulate micellar volume and the particle-size distribution in polymeric systems.

Micelle size is a critical parameter, influenced by the materials used and the intended application. In the pharmaceutical context, smaller particles enhance cellular uptake and permeability [54,55]. For agricultural applications in pest control, particles ranging from 28 to 559 nm remain bioactive [56,57], and diameters up to 500 nm are generally considered suitable [58,59]. In the context of insecticidal formulations, particle sizes below 500 nm are considered appropriate. This size range provides desirable physicochemical properties, including greater surface area, colloidal stability, and dispersion efficiency, facilitating absorption and/or adhesion to the body of the target insect, enhancing insecticidal action. Consequently, selecting particles within this range supports both bioactivity and overall viability of the proposed approach.

#### 2.2.2. Accelerated Stability Assay of the Formulations

The heat stress stability test is important for evaluating a formulation’s ability to resist temperature variations without compromising its macroscopic and microscopic characteristics. The test aims to detect any changes induced by the heating-cooling cycle in the formulation. Only minimal variations in hydrodynamic diameter and polydispersity index (PDI) were observed (Table S1), indicating good micellar stability. Similar thermal stress protocols have been widely employed to assess the physical stability of polymeric formulations [28,60].

Formulations prepared with Pluronic^®^ F-127 exhibited temperature-dependent behavior, as evidenced by variations in hydrodynamic diameter. Dynamic light scattering (DLS) assays conducted between 4 °C and 45 °C have demonstrated that PF-127 nanomicelles can undergo more than 200-fold decrease in hydrodynamic diameter upon heating [61]. This phenomenon is associated with the PEO_100_–PPO_65_–PEO_100_ architecture of the copolymer, where progressive dehydration of the increasingly hydrophobic PEO blocks leads to the expulsion of water molecules micellar volume compression [61,62,63]. Similar behavior has been reported in systems containing PF-127 combined with polyethylenimine, heparin, or chitosan [64,65,66,67].

**Figure 3:**
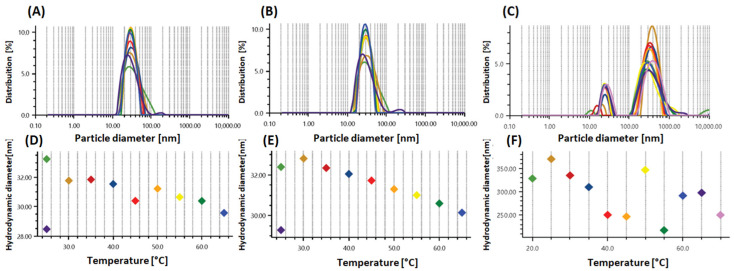
Particle size distribution of the formulations PEOANIB (**A**), PNFTANE (**B**) and PNFTENE (**C**). Variation in hydrodynamic diameter (y) versus temperature variation (x): PEOANIB (**D**), PNFTANE (**E**) and PNFTENE (**F**).

In the present study, the inclusion of DMSO as a co-solvent reduced the thermal sensitivity of the system as the particle-size distribution curves remained virtually unchanged (Figure 3). Thermal stress assays confirmed the structural integrity of PEOANIB, PNFTANE, and PNFTENE up to 60 °C (Table 4). For the PNFTENE formulation, the initial PDI was 0.206, with approximately 5% of particles at 20.6 nm (25 °C), while 95% were concentrated around 399 nm. After the 25 → 60 → 25 °C thermal cycle, the main diameter decreased to 321.9 nm, the minor peak reduced to 1.1% of the total area, and the PDI increased slightly to 0.236, indicating minor variations without loss of stability. This contraction is attributed to the structural reorganization of the copolymer, which promotes the progressive desolvation/dehydration of the PPO blocks. This process increases chain hydrophobicity with rising temperature, resulting in compaction and a consequent reduction in the micellar volume [28,61,68].

Analysis of the mean, standard deviation, and RSD% (Table S1) revealed an increase in the fraction of micelles smaller than 30 nm, without compromising overall stability. Light transmittance varied by only 0.31%. Despite changes of up to 20.9 percent in PDI and 18.3 percent in particle diameter across 25–65 °C, the visual appearance remained unchanged (Figure 3). The slight increase in polydispersity reflects the formation of micellar subpopulations, a phenomenon related to the critical polymer concentration required for system stabilization [69]. These results indicate that the formulations remain stable up to 60 °C; however, the optimal storage range is 25–35 °C, which preserves the original particle-size distribution and PDI values, thus providing crucial information for the storage and future handling of these formulations.

#### 2.2.3. Room Temperature Stability Assay of PEOANIB, PNFTANE and PFTENO Formulations

At room temperature, PEOANIB and PNFTANE remained physically stable for 120 days, with no significant changes in PDI, hydrodynamic diameter, or transmittance (Table S2). Over this period, mean particle sizes were 30.7 ± 2.7 nm (relative standard deviation, RSD, 8.7%) for PEOANIB and 32.0 ± 1.3 nm (RSD 4.1%) for PNFTANE. Corresponding PDI values were 0.164 ± 0.051 (RSD 31%) and 0.198 ± 0.025 (RSD 12.8%), respectively. For the PNFTENE formulation, monitored for 190 days, the mean particle size was 374 ± 36.4 nm (RSD 9.7%) and the mean PDI was 0.209 ± 0.054 (RSD 25.8%). All formulations exhibited low size variability, PDI below 0.30, and consistent transmittance, supporting sustained macroscopic and microscopic stability throughout the evaluation period [28,60]. Valenzuela-Oses and collaborators showed that raising the sample concentration from 6 to 12 mM led to a bimodal micellar distribution [70]. In line with this, adding NFTENE to the formulation generated a bimodal particle-size distribution, suggesting that the concentration of loaded compounds shapes the micellar profile (Table S2).

### 2.3. Larvicidal Activity

For the larvicidal studies, EOANIB, NFTANE, NFTENE and their respective formulations (PEOANIB, PNFTANE and PNFTENE) were evaluated. EOANIB showed significant larvicidal activity against third instar larvae (L3) of *Aedes aegypti*, with LC_50_ of 86.9 ppm at 24 h and 84.3 ppm at 48 h, with no significant difference between time points (*p* > 0.05). NFTANE displayed comparable potency (24 h LC_50_ of 85.8 ppm; 48 h LC_50_ = 83.6 ppm). The lack of a significant difference between EOANIB and its major constituent NFTANE supports the view that NFTANE is a principal determinant of the insecticidal effect of the essential oil.

The structural modification of NFTANE into the synthetic derivative NFTENE, involving the introduction of a double bond between the C1–C2 atoms of carbon (Figure 1), resulted in a substantial enhancement of biological activity. NFTENE exhibited an LC_50_ of 7.7 ppm after 48 h, approximately tenfold more potent than NFTANE (83.6 ppm) (Table 3). This effect may be attributed to the conformational restriction imposed by the double bond, which maintains the nitro group in a trans configuration, reducing molecular flexibility and potentially optimizing interactions with biological targets. Previous studies support this hypothesis, demonstrating that physicochemical properties such as molecular weight, partition coefficient (LogP), and rotational restriction significantly influence the biological activity of drug-like molecules [23,24,25,26,27].

Encapsulation in polymeric nanomicelles markedly enhanced larvicidal potency. PEOANIB and PNFTANE reduced 48 h LC_50_ values to 50.5 ppm and 47.2 ppm, corresponding to 40% and 43% decreases relative to the corresponding free compounds (Table 4). An even larger effect was obtained with PNFTENE (LC_50_ of 4.4 ppm), a 42% reduction relative to NFTENE diluted in DMSO. These findings are particularly relevant from an environmental perspective, demonstrating the potential to reduce the amount of insecticide required for effective vector control, aligning with principles of sustainable vector management [28,71,72]. Pluronic^®^ F-127 offers advantages due to its low toxicity. Comparative studies of F68, F108, and F127 copolymers revealed that F127 exhibits the lowest hemolytic activity in erythrocytes and allows safe intravenous administration of formulations containing up to 4.5% F127 [73]. Its biocompatibility, biodegradability, and structural similarity to the extracellular matrix make F127 a promising polymeric vehicle for drug and bioactive compound delivery [74,75].

Biological activity stability tests at fixed concentrations (70 ppm for PNFTANE and 5 ppm for PNFTENE) confirmed sustained larvicidal efficacy for up to 14 days. PNFTANE maintained stable larval mortality (82.5 ± 2.9% at baseline versus 88.1 ± 6.5% on day 14), while PNFTENE showed a nonsignificant change of 17.6 ± 11.9% over the same interval (*p* > 0.05). A *t* test detected no significant differences between replicates, supporting the structural and biological stability of the formulations.

Operationally, LC_50_ thresholds are often defined as >100 ppm (weak or inactive), 50–100 ppm (active), and <50 ppm (highly active) [71,76,77,78]. In this study, all tested samples showed LC_50_ < 100 ppm against *Aedes aegypti*: EOANIB and NFTANE were in the active range (84–87 ppm), whereas PNFTANE and PNFTENE fell within the highly active range (47.2 ppm and 4.4 ppm, respectively). While many essential oils exhibit larvicidal effects, comparatively few reports LC_50_ values below 50 ppm [71,76,77,78]. For example, essential oils from *Guatteria hispida* and *Eugenia melanadenia* show LC_50_ of 85 ppm against *A. aegypti* larvae [71], comparable to the free oil and constituent reported here.

### 2.4. Adulticidal Activity

The data presented in Table 4 demonstrate that EOANIB exhibits adulticidal activity against *Aedes aegypti* females, with time-dependent variations in efficacy. The lowest LC_50_ value was achieved after 90 min of exposure at 33.9 ppm, indicating the highest potency at this time point. However, mortality observed after 24 h was not statistically different (*p* > 0.05) from that recorded at 45 min. Considering efficacy and practical exposure time, 45 min is an optimal exposure period, achieving an LC_50_ of 43.5 ppm. According to the CDC manual, the diagnostic dose was found at LC_99.9_ = 119.0 (101.9 to 150.1) ppm and the diagnostic time at 45 min [79].

When compared to previous studies using essential oils for *A. aegypti* control, the results obtained with EOANIB appear significantly superior. França and coworkers reported LC_50_ and LC_90_ values of 135.76 ppm and 243.76 ppm, respectively, for the essential oil of *Piper capitarianum* leaves [80], while *Piper aduncum* showed LC_50_ and LC_90_ values of 401 ppm and 492 ppm [81]. Essential oils from other species such as *Croton linearis* (LC_50_ = 79.9 ppm), *Lantana involucrata* (LC_50_ = 107.8 ppm), and *Ocimum sanctum* (LC_50_ = 119.8 ppm) displayed comparable potency compared to EOANIB [82].

The biological efficacy of essential oils is closely associated with their phytochemical composition [81]. Previous studies have reported adulticidal activity for minor constituents present in EOANIB, such as α-pinene (LC_50_ = 17.3–56.5 ppm), α-caryophyllene, and β-caryophyllene (LC_50_ = 26 ppm) [77], while 2-phenylacetaldehyde, known for its insect-attractant properties [83], does not affect the insecticidal under the conditions tested. These results support the hypothesis that the major constituent of EOANIB is primarily responsible for the bioactivity observed.

NFTANE accounted for 70% of EOANIB and has been associated with acetylcholinesterase (AChE) inhibition [20,21]. Together with the absence of a significant difference between the insecticidal activities of EOANIB and isolated NFTANE, these data support the hypothesis that NFTANE is the primary contributor to toxicity in both larval and adult *Aedes aegypti*, plausibly via AChE inhibition. These results support the development of sustainable formulations based on the leaf essential oil of *A*. *canelilla*, a renewable resource available year-round and more abundant during the Amazon dry season [19]. Moreover, the larvicidal and adulticidal assays indicate that *Aniba canelilla* can target multiple stages of the mosquito life cycle, enhancing its applicability in integrated vector management strategies. This potential is particularly relevant given the limitations of synthetic insecticides, which pose high toxicity risks to non-target organisms and human health, contributing to the emergence of resistant mosquito populations [13,15,77].

Adulticidal activity was evaluated exclusively for EOANIB due to methodological constraints. The CDC bottle bioassay requires impregnation of the bottle surface, which is incompatible with aqueous nanoformulations (PEOANIB, PNFTANE, PNFTENE). Preliminary tests with isolated NFTANE showed comparable adulticidal effects to EOANIB, consistent with larvicidal assay results. In contrast, NFTENE exhibited no adult mortality at 100 ppm. This lack of activity is likely related to its physicochemical characteristics, as NFTENE is a crystalline, non-volatile compound, unlike the volatile EOANIB, which can effectively interact with the insect respiratory system. For these reasons, additional adulticidal tests for NFTENE and the nanoformulated samples were not conducted, as their physical nature is incompatible with the assay protocol.

Although several studies have explored the use of essential oils against *A. aegypti*, the present work is, to our knowledge, the first to show that *A. canelilla* leaf essential oil is active against both larvae and adults, providing a novel and promising option for direct control [71,77,84,85].

### 2.5. Toxicity to the Non-Target Insect (Artemia salina)

According to the criterion of Meyer and coworkers, which sets LC_50_ < 1000 ppm as the toxicity threshold, EOANIB, NFTANE, and NFTENE fall within this category (Table 5) [86]. NFTENE was the most potent, with an LC_50_ of 1.9 ppm. Nevertheless, the observed LC_50_ lies within the range reported for representative synthetic insecticides, including profenofos^®^ (7.71 ppm) and chlorpyrifos^®^ (0.38 ppm) [87]. In the assays performed with PNFTENE at concentrations up to 4.0 ppm, no mortality of *Artemia salina* nauplii was observed. This lack of effect may be associated with particle size (370 nm) being insufficient to permeate the microcrustacean membranes, the salinity of the assay medium, or differences in membrane chemical composition that could limit nanomicelle diffusion [88]. Notably, the nanoformulations PEOANIB and PNFTANE demonstrated substantially lower toxicity to *A. salina* (LC_50_ = 82.0 and 91.2 ppm, respectively) compared to conventional synthetic insecticides [87,89], representing an approximately 11- to 240-fold reduction in toxicity to non-target organisms. This favorable toxicological profile is consistent with data reported for other plant-derived products, which have demonstrated LC_50_ values ranging from 13 to 144.75 ppm against *A. salina* [90,91].

### 2.6. Toxicity to the Non-Target Insect (Drosophila melanogaster)

**Figure 4:**
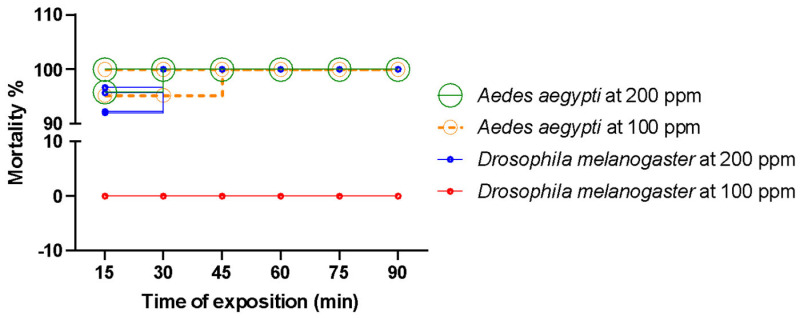
Percentage mortality (%) of *Aedes aegypti* and *Drosophila melanogaster* at different test concentrations of EOANIB. Control groups treated with acetone showed no mortality in either species. Post-transfer conditions temperature 27 ± 1 °C, Relative humidity 70 ± 5%.

Selectivity assays using *Drosophila melanogaster* revealed a toxicity profile distinct from that observed in *Aedes aegypti*. At a concentration of 200 ppm, 100% mortality was observed in *D. melanogaster* after 90 min of exposure, whereas no mortality was detected at 100 ppm. In contrast, *A. aegypti* exhibited 100% mortality at both tested concentrations (Figure 4).

The observed selectivity may result from physiological and biochemical differences between the species. *D. melanogaster* possesses highly active detoxification pathways, including cytochrome P450 enzymes, glutathione S-transferases, and carboxylesterases, which provide a greater capacity for xenobiotic metabolism when compared to *A. aegypti* [23,92]. Additionally, variations in the catalytic site structure of acetylcholinesterase (AChE) may influence species-specific susceptibility to the tested compounds [93].

Assessing toxicity in non-target organisms is essential for evaluating the selectivity of insecticides. Evaluating effects on non-target species helps identify essential oils and compounds with high specificity for the target insect, minimizing unintended impacts on ecologically beneficial or neutral organisms. This approach supports the development of safer control strategies by reducing potential toxic effects on other organisms and minimizing the risk of ecological imbalances [94]. *D. melanogaster* is a suitable model organism for such studies due to its ecological relevance in ecosystems and the extensive knowledge of its physiology [95,96,97]. Experimental results obtained in this work show that concentrations of approximately 100 ppm achieve high efficacy against *Aedes aegypti* with lower toxicity toward *Drosophila melanogaster*, consistent with selective use in vector control strategies.

### 2.7. Acetylcholinesterase (AChE) Inhibition Activity

Acetylcholinesterase inhibition is a key endpoint for clarifying the mode of action of insecticidal agents against disease-vector mosquitoes. The inhibitory potential of *Aniba canelilla* leaf essential oil, NFTANE and NFTENE was evaluated in a dose-dependent manner as a plausible mechanism contributing to their insecticidal activity. Assays were conducted with two enzyme sources, purified acetylcholinesterase from *Electrophorus electricus* and insect homogenates. The purified enzyme provides standardized, reproducible baseline measurements, whereas homogenates provide a biologically relevant matrix that incorporates potential cofactors, endogenous inhibitors, and metabolic components that may modulate the observed inhibition. This dual approach allows comparison between highly controlled and physiologically complex conditions.

#### 2.7.1. Inhibitory Activity on Commercial Enzyme (*Electrophorus electricus*)

Initial validation confirmed enzyme activity reliability. The commercial enzyme was diluted to 150 mU, exhibited an activity of 152 mU, corresponding to less than 5% error with excellent linearity (R^2^ = 0.9924). All tested samples exhibited significant inhibitory activity against commercial AChe, with IC_50_ values of 163.2 (149.2–178.1) µg∙mL^−1^ for EOANIB, 189.7 (171.6–209.4) µg/mL for NFTANE, and 148.9 (131.7–167.4) µg∙mL^−1^ for NFTENE (Table 6). Among them, NFTENE was the most potent inhibitor, followed by EOANIB and NFTANE. Duque and collaborators demonstrated that complex biological matrices significantly reduce inhibition efficiency, with commercial enzyme inhibition ranging from 22.7% to 55.7% compared to 12.6% to 37.7% in larval homogenates [98].

#### 2.7.2. Inhibitory Activity in Insect Homogenates

**Figure 5:**
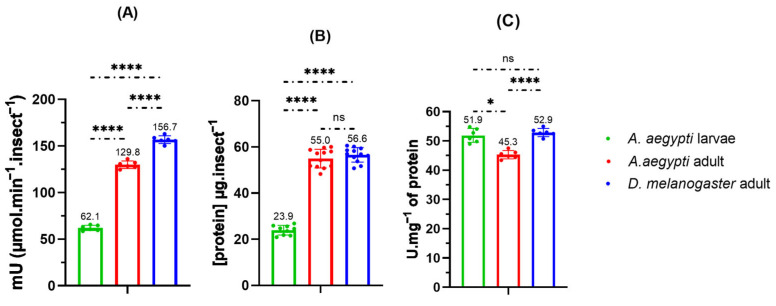
Enzyme activity, protein amounts, specific activities in *Aedes aegypti* larvae and adults and *Drosophila melanogaster* adults. Amount of acetylcholinesterase enzyme per insect (**A**), Amount of total proteins/insect (**B**), Specific activity (**C**). Samples were subjected to analysis of variance (ANOVA) followed by unpaired Tukey’s test. * (*p* value < 0.05), **** (*p* value < 0.0001), ns (*p* value > 0.05). Positive controls were performed with the commercial enzyme from *Electrophorus electricus*.

Prior to performing the enzymatic inhibition experiments, protein content in insect homogenates of *Aedes aegypti* and *Drosophila melanogaster* was quantified to estimate the specific activity (U mg^−1^) of AChE. The assays showed that *D. melanogaster* expresses approximately 53.0 U of acetylcholinesterase per milligram of total protein, whereas *A. aegypti* adults and larvae exhibit 45.3 and 51.9.1 U mg^−1^, respectively (Figure 5).

**Figure 6:**
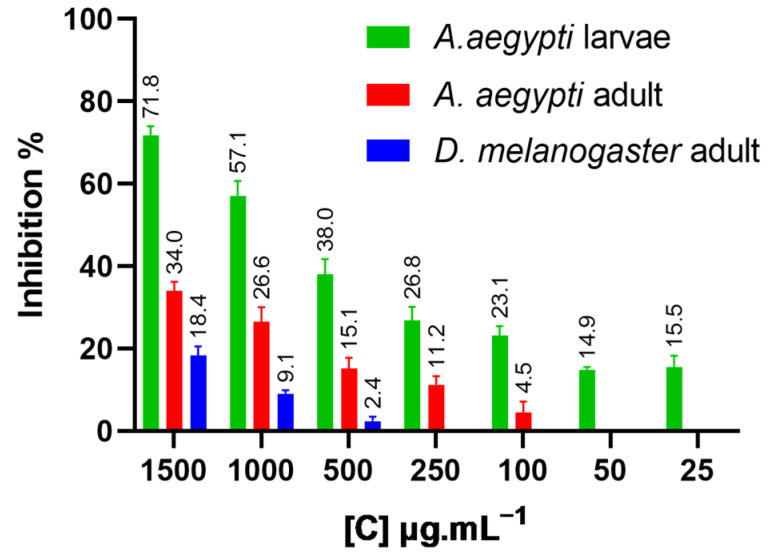
Percentage inhibition of the acetylcholinesterase enzyme using NFTENE in concentrations in µg/mL compared to the homogenate of larvae (Green) and adults (red) of *Aedes aegypti* and *Drosophila melanogaster* flies (blue).

The specific activity measured in *D. melanogaster* (53.0 U∙mg^−1^) exceeds the 0.3 U∙mg^−1^ reported by Gnagey and collaborators for head only extracts [93], consistent with the use of whole body homogenates here versus head preparations in the prior work. Body composition differed across species and stages: *A. aegypti* larvae averaged 1480 µg total mass and 23.9 µg protein (1.6%), adults 655 µg and 55.0 µg (8.4%), and *D. melanogaster* adults 892 µg and 56.6 µg (6.3%). Despite similar protein content between adult species, *D. melanogaster* was less susceptible to the tested essential oil and derivatives. In homogenate assays, EOANIB and NFTANE produced ≤10% inhibition at 1500 µg mL^−1^, whereas NFTENE yielded 71.8% inhibition in *A. aegypti* L3 larvae, 34.0% in adults, and 18.4% in *D. melanogaster* adults (Table 7 and Figure 6). Given that higher acetylcholinesterase concentrations correlate with increased resistance to AChE-inhibiting insecticides [99], the interspecific enzymatic profile supports the potential selectivity of *A. canelilla* essential oil for *A. aegypti* control.

These detoxification pathways involve enzymatic modification of toxic compounds through oxidation, hydrolysis, and conjugation reactions, potentially reducing inhibitor bioavailability and efficacy [92]. Such enzymatic background explains the differential activity between the purified enzyme and complex biological matrices [98,100].

The results indicate that acetylcholinesterase inhibition is a relevant mode of action, particularly for NFTENE against *Aedes aegypti* larvae. This molecular insight helps explain the high efficacy observed and supports the development of targeted vector control strategies based on *A. canelilla* essential oil.

## 3. Materials and Methods

### 3.1. Reagents and Chemicals

Acetylcholinesterase (AChE) from *Electrophorus electricus* (C3389-2KU), acetylcholine iodide (AChI), 5,5′-dithiobis-2-nitrobenzoic acid (DTNB), Pluronic^®^ F127 ((PEO)100-(PPO)65-(PEO)100; Mw: 12,600 Da), phosphate-buffered sodium, galantamine hydrobromide were purchased from Prati-Donizetti Laboratories, 1-Nitro-2-phenylethene (NFTENE) was obtained from Sigma-Aldrich Corporation (St. Louis, MO, USA). Temephos Abate^®^ insecticide was purchased from BASF, Malaysia. K-Othrine^®^ SC 25 was purchased from (Bayer Crop. Sciences, Frankfurt am Main, Germany). Distilled water was used for general procedures. Solvents dichloromethane, dimethyl sulfoxide (DMSO), acetone and methanol were of analytical and chromatographic grades (TEDIA).

### 3.2. Plant Material

Leaves of *Aniba canelilla* (H.B.K.) Mez were collected at the Duke Reserve, Manaus, Amazonas state. A voucher specimen (no. 220094) was deposited in the Herbarium of the National Institute of Amazonian Research (INPA). The leaves were air-dried under shade.

### 3.3. Obtention of the Essential Oil from Aniba canelilla (EOANIB) and Isolation of 1-Nitro-2-Phenylethane (NFTANE)

The essential oil was obtained by hydrodistillation using a Clevenger-type apparatus, employing approximately 496 g of dried and fragmented leaves of *Aniba canelilla*. The process was carried out for 2 h, and the resulting essential oil was stored at −10 °C in amber glass vials to preserve its chemical composition.

For the isolation of the major compound, 1-Nitro-2-phenylethane (NFTANE), 1.464 g of essential oil were initially dissolved in 1200 μL of dichloromethane and subjected to distillation in a Kugelrohr microdistillation system (Büchi Glass Oven B-585), operated at 110 °C and 200 mBar. The system consisted of three interconnected flasks, in which the sample was placed in the innermost flask and maintained under heating, while the purified product was collected in the outermost flask containing a cooling mixture of dry ice and ethanol.

### 3.4. GC–MS Analysis of Samples

GC–MS analyses were carried out using a Shimadzu (Kyoto, Japan) GC-2010 Plus gas chromatograph coupled to a QP-2010 selective mass detector operating under electron impact ionization (70 eV). The chromatograph was equipped with a non-polar capillary column DB-5MS (30 m × 0.25 mm i.d., film thickness 0.25 μm). Helium was used as the carrier gas at a constant flow rate of 0.5 mL/min. The oven temperature program was as follows: initial temperature of 40 °C, raised to 300 °C at a rate of 4 °C/min, followed by an isothermal hold at 300 °C for 10 min. Injector and detector temperatures were maintained at 250 °C. The injection volume was 1.0 μL (2 mg/mL in CH_2_Cl_2_) in splitless mode. The linear velocity (ū) was set at 14 cm/s. The MS interface temperature was set at 280 °C. Mass spectra were acquired over a mass range of 40–700 *m*/*z*, with a scan speed of 150 amu/s and acquisition interval of 0.50 s (2 Hz). Volatile constituents were identified by comparing their mass spectra and retention indices with those reported in the literature or available in the Wiley mass spectral database associated with the GC–MS system.

### 3.5. Preparation of Micelles with Pluronic^®^ F-127 (PF-127)

Micelles were prepared according to the procedure using the polymeric film hydration method reported by [44], with minor modifications. For the formulations containing the essential oil EOANIB and its major constituent NFTANE whose chemical structure is shown in Figure 1A, 200 mg of the copolymer PF-127 (5.0% *w*/*v*) were weighed, followed by approximately 60 mg of the active material (1.5% *w*/*v*) and 100 µL of DMSO (112.5 mg) as a co-solvent.

For the formulation with the synthetic derivative NFTENE (structure in Figure 1B), the same amount of copolymer was used (200 mg; 5.0% *w*/*v*), together with 13.5 mg of the active compound (0.34% *w*/*v*) and an equivalent volume of DMSO (100 µL; 112.5 mg; 2.4% *v*/*v*). Exact quantities for all components are provided in Table 8.

Masses, volumes, and theoretical final concentrations are summarized in Table 1. The final concentrations of PEOANIB, PNFTANE, and PNFTENE were 14.75 mg mL^−1^, 14.43 mg mL^−1^, and 3.37 mg mL^−1^, respectively. A blank formulation (PBlank) was included in the larvicidal assays to assess any potential effects of the unloaded polymeric matrix on larval mortality.

The preparation process began with dissolution of the components in 2 mL of dichloromethane (CH_2_Cl_2_), followed by vortex homogenization at 800 rpm for 2 min. The organic solvent was then completely removed with a rotary evaporator. Subsequently, 4 mL of ultrapure water (25 °C) were added to the mixture, and homogenization was carried out with an IKA Ultra-Turrax^®^ (model T10BS32, Staufen, Germany) at speed setting 5 for 5 min. The resulting micelles were subjected to ultrasonic treatment in a Unique UltraSonic^®^ bath (model USC-1800A, São Paulo, Brazil) at 40 kHz for 5 min to reduce particle size and stabilize the system. After sonication, the formulations were filtered through a 0.45 µm regenerated cellulose (RC) hydrophilic syringe filter.

#### 3.5.1. Micelle Evaluation

PF-127 micelles containing the active compounds EOANIB and NFTANE (1.25%) and NFTENE (0.34%) were physicochemically characterized for particle size, polydispersity index (PDI) and zeta potential. Samples were diluted 1:20 (*v*/*v*) in Milli-Q^®^ water before analysis. Measurements were performed by dynamic light scattering (DLS) using an Anton Paar Litesizer^®^ 500 (Graz, Austria) at 25 °C with a scattering angle fixed of 90°.

The zeta potential analysis method was performed at a fixed voltage of 200 V at a temperature of 25 °C, scattering angle fixed at 90° and waiting time between analyses of 2 min using the Smoluchowski adjustment for the calculations as a parameter.

#### 3.5.2. Stability Evaluation Under Thermal Stress and at Room Temperature

Stability studies employed the same formulation batch prepared as described in Section 2.5. For thermal-stress assessment, analyses were conducted 24 h after preparation with the Anton Paar Litesizer^TM^ 500. Samples diluted 1:20 (*v*/*v*) in ultrapure water were exposed to a controlled temperature gradient from 25.0 ± 0.1 °C to 65.0 ± 0.1 °C in 5.0 ± 0.1 °C increments, with an equilibration time of 3 min at each step. In parallel, formulations stored in sealed vials under ambient conditions (25 ± 2 °C) were monitored for up to 120 days for PEOANIB and PNFTANE and up to 190 days for PNFTENE [28,101].

Room-temperature stability analyses were performed immediately after preparation and at regular intervals during storage, using the same dilution protocol and equipment as for the thermal-stress tests. Parameters monitored included mean particle size (nm), PDI, zeta potential (mV) and transmittance (T %). Macroscopic attributes of homogeneity, phase separation, sedimentation and creaming were also inspected. All assays were conducted in triplicate with independent batches prepared under identical conditions, as detailed by [28,102].

### 3.6. Larvicidal Activity Against Aedes aegypti

Eggs of the Rockefeller strain of *Aedes aegypti* were hatched in filtered water, and larvae were reared under controlled conditions (28 ± 1 °C; 12 h photoperiod) until reaching the third instar (L3). Bioassays followed an adapted protocol in 50 mL glass vessels, each containing 20 mL of filtered water and 20 size-matched third instar (L3) larvae [103,104]. Each test condition, including controls, was run in quadruplicate. Test solutions were prepared as follows: EOANIB and NFTANE, 150 µg mL^−1^ to 5 µg mL^−1^; NFTENE, 25 µg mL^−1^ to 0.1 µg mL^−1^. Formulations (PEOANIB, PNFTANE, PNFTENE) were applied with the same protocol used for DMSO-dissolved samples, keeping the final solvent concentration at 1% (*v*/*v*) of the total water volume.

Negative controls contained either DMSO (200 µL) alone or blank nanomicelles (NBr) at a volume matching the highest concentration tested, whereas the positive control comprised temephos at 1 µg mL^−1^. After 14 days of preparation of the formulation, an activity confirmation test was performed at fixed concentrations of PNFTANE (70 ppm) and PNFTENE (5 ppm) formulations. The assays were performed in quadruplicate.

### 3.7. Adulticidal Activity Against Aedes aegypti

*A. aegypti* colony was maintained at 26 ± 1 °C, 80 ± 5% relative humidity, and a 12 h light–dark cycle. Bioassays followed the WHO bottle protocol for assessing insecticide resistance in vectors WHO, with adaptations based on the U.S. CDC method [79,105]. Glass bottles (295 mL) were coated with 1 mL of essential oil solution in analytical-grade acetone. Bottles were left open for 10 min to allow complete solvent evaporation, then acclimated for 1 h with the cap removed [106]. Each bottle received 15–25 unfed adult female *A. aegypti* and was exposed to 200–50 ppm of the test compound. Deltamethrin (K-Othrine^®^) at 10 ppm served as the positive control, and acetone-treated bottles as the negative control. Assays were conducted in quadruplicate, with mortality recorded at 15 min intervals over 90 min of continuous exposure. After 90 min, mosquitoes were gently transferred to 500 mL cylindrical paper containers covered with nylon mesh and kept under colony conditions (26 °C, 80% RH, 12 h photoperiod) for knockdown assessment 24 h post-exposure.

### 3.8. Non-Target Organism Assay

#### 3.8.1. Non-Target Toxicity (*Artemia salina*)

Toxicity assays with *Artemia salina* followed the procedure of Meyer and collaborators with adaptations like those of [86,107]. For cyst hatching, 25 mg of eggs were placed in a cuboid-glass vessel containing 600 mL of artificial seawater (38 g L^−1^ in distilled water) and incubated at 28–30 °C under continuous illumination from a 40 W incandescent lamp. After 24 h, the nauplii (first larval stage) were transferred to fresh saline and reared for an additional 24 h under the same conditions to obtain metanauplii (second larval stage), which were used in the bioassays. Ten metanauplii were exposed to 10 mL of saline containing the test samples dissolved in DMSO. EOANIB, PEOANIB, NFTANE, PNFTANE, NFTENE and PNFTENE were assayed at seven concentrations (1000–15.6 µg mL^−1^), whereas NFTENE previously shown to be more toxic in larvicidal test was evaluated at eight concentrations (125–0.6 µg mL^−1^). Stock solutions were prepared at 50 mg mL^−1^ (EOANIB, NFTANE) and 6.25 mg mL^−1^ (NFTENE); application volumes (200–3.12 µL) were adjusted so that the final DMSO content never exceeded 1% of the total volume. After dosing, organisms were maintained at 28–30 °C under artificial light for 24 h. Mortality defined as immobility or sinking to the bottom was recorded, and LC_50_ values were calculated for each sample. All experiments were performed in triplicate with independent replicates under identical conditions to ensure reproducibility.

#### 3.8.2. Non-Target Organism Toxicity (Drosophila melanogaster)

*Drosophila melanogaster* colony was maintained at 30 ± 1 °C, 80 ± 5% relative humidity and a 12 h light–dark cycle. Toxicity was assessed with a modified version of the CDC bottle assay originally developed for insecticide-resistance testing in vectors [79]. Glass bottles (295 mL) were coated with 1 mL of the essential-oil solution in analytical grade acetone (Merck). Bottles remained open for 10 min to allow complete solvent evaporation, followed by 1 h acclimation without caps [106]. Each bottle received 25–30 unsexed adult flies and was challenged with 300–50 ppm of the test sample. Acetone treated bottles as negative controls, whereas deltamethrin K-Othrine^®^ at 10 ppm was the positive control. All assays were conducted in quadruplicate, and mortality was recorded every 15 min during 90 min of continuous exposure.

### 3.9. Enzymatic Assays

#### 3.9.1. Preparation of *Aedes aegypti* (Adults and L_3_ Larvae) and *Drosophila melanogaster* Adult Homogenates

A total of 250 third instar larvae (370.1 mg) and 250 adults (163.8 mg) of *A. aegypti*, together with 150 adults of *D. melanogaster* (134.6 mg), were collected. Each group was placed in a separate 50 mL Falcon tube and frozen at −20 °C to arrest metabolic activity. After freezing, 5 mL of ultrapure water were added to each tube, and the contents were mechanically homogenized on ice using an IKA Ultra-Turrax^®^ homogenizer (model T10BS32) at speed setting 4 for 5 min. The resulting homogenates were transferred to centrifuge tubes and spun at 10,000 rpm for 5 min at 4 °C in a refrigerated centrifuge. Supernatants were carefully collected, aliquoted and stored at −20 °C for biochemical analyses performed within 24 h [107]. After preparation, the homogenates contained 50 insects/mL (larvae) and 30 insects/mL (adult *Aedes aegypti* and *Drosophila melanogaster*). These preparations were subsequently used for acetylcholinesterase inhibition activity, total-protein quantification by the BCA method, and calculation of specific catalytic units per individual.

#### 3.9.2. Total Protein, Catalytic Units (µmol∙min^−1^), and Specific Activity

Total protein was quantified with the Pierce^TM^ BCA Protein Assay Kit (Thermo Scientific^TM^, REF 23225, Rockford, IL, USA) according to the manufacturer’s instructions, with slight modifications. Working reagent was prepared by mixing 9.8 mL of Reagent A with 0.2 mL of Reagent B. A BSA standard curve (0.1–2.0 µg µL^−1^) was generated from a 2 mg mL^−1^ stock solution. Reactions were set up in 96-well plates with 180 µL working reagent and 20 µL of either standards or homogenates diluted 1:4 in ultrapure water. Plates were incubated at 37 °C for 30 min in the dark, and absorbance was measured at 562 nm on the SpectraMax^®^ 190 (Molecular Devices, Sunnyvale, CA, USA). Specific catalytic units were determined using a 2-mercaptoethanol calibration curve. A 5.0 mM stock (39 mg in methanol, diluted to 100 mL) was serially diluted to 1–5000 µM. Each well received 160 µL of 0.1 M phosphate buffer (pH 8.0), 20 µL of 3.0 mM DTNB, and 20 µL of standard solution. After gentle mixing, end-point absorbance was read at 405 nm with the same microplate reader. Sample absorbance values were interpolated on the 2-mercaptoethanol curve to obtain product formed (µmol min^−1^), which was then normalized to total protein content from the BCA assay. Results are reported as specific catalytic units per insect (µmol min^−1^ mg^−1^ protein) [93].

#### 3.9.3. Acetylcholinesterase (AChE) Inhibition Assay

Percentage inhibition of AChE was determined by the standard Ellman colorimetric method, with minor adaptations [108]. In each well of a 96-well microplate, the following were added in sequence: 120 µL of 0.1 M phosphate buffer (pH 8.0), 20 µL AChE (*E. electricus*, 0.15 U mL^−1^) or insect homogenate and 20 µL of EOANIB, NFTANE and NFTENE inhibitor solution in methanol (25–1500 µg mL^−1^). After a 10 min incubation at 30 °C, 20 µL of DTNB (3.0 mM) and 20 µL of acetylthiocholine iodide (10.0 mM) were added. Plates were then incubated for a further 30 min at 30 °C, and end-point absorbance was read at 405 nm and pathlength 0.62 cm on a SpectraMax^®^ 190 microplate reader (Molecular Devices^TM^, SoftMax^®^ Pro 5; serial no. SMP500-11007-TIQG).

### 3.10. Statistical Analysis

The lethal concentrations of 50%. 90%, 99% and 99,9% of the insects (LC_50_; LC_90_; LC_99_; LC_99.9_) with 95% confidence limits were determined by probit regression of the replicate means of each concentration using STATGRAPHICS Centurion XV Version 15.1.02. Additionally, the χ^2^ for each probit model was calculated with the same software [109]. Significant differences were determined by analysis of variance (bidirectional ANOVA) followed by Tukey’s test (*p* < 0.05). Significant differences were determined by the Wilcoxon test for non-target *Artemia salina* and valor enzymatic activity. IC_50_ values (50% inhibitory concentration) were calculated using the software GraphPad Prism 9.0.2. (GraphPad, CA, USA).

## 4. Conclusions

The dataset presents a coherent case for *Aniba canelilla* as a botanical source for *Aedes aegypti* control, with activity demonstrated against both larval and adult stages. The incorporation of these compounds into PF-127-based polymeric nanomicelles significantly enhanced their biological activity and provided adequate stability for practical applications. Selectivity assays revealed distinct toxicological profiles between target and non-target organisms, suggesting potential for selective application in vector control. The elucidation of the mode of action via acetylcholinesterase inhibition contributes to a deeper understanding of the molecular basis underlying insecticidal efficacy. The results highlight the potential of *A. canelilla* for *A. aegypti* control, broadening the repertoire of plant-derived agents. Overall, the work expands the repertoire of botanical options with a plausible mode of action and a formulation route that improves stability and activity, while highlighting clear next steps to move from laboratory promise to deployable vector-control tools.

## Figures and Tables

**Table 1:** GC–MS composition of hydrodistilled *Aniba canelilla* leaf essential oil (EOANIB).

Compounds *	RT (min)	RI_L_	EOANIB (% Area)
α-pinene	12.9	932	1.4
benzene acetaldehyde	17.8	1036	4.2
benzene acetonitrile	21.9	1134	0.6
1-Nitro-2-phenylethane	28.0	1350	70.0
β-caryophyllene	31.0	1417	2.2
α-caryophyllene	31.5	1452	1.7
β-bisabolene	34.2	1505	1.1
(E)-nerolidol	38.7	1561	3.2
% Total			84.4

*: based on Willey library and Adams (2007) [34]; RT: retention time; RI_L_: literature retention index [33].

**Table 2:** Dynamic Light Scattering (DLS) analysis of formulations PEOANIB, PNFTANE, and PNFTENE.

Sample	PS (nm)	Area %	PDI	T%	IZP (mV) ± SD	FZP (mV) ± SD
PEOANIB	33.4 ± 12.8	100	0.20	87.2	−11.4 ± 0.9	−6.3 ± 1.6 *
PNFTANE	32.4 ± 3.0	100	0.15	86.3	−23.1 ± 1.1	−14.4 ± 2.4 *
PNFTENE	370 ± 63	95.1	0.13	77.5	−17.8 ± 0.6	−16.6 ± 0.1 **

All samples were diluted 1:20 (*v*/*v*) with water before analysis. Parameters evaluated include particle size, nm (PS), polydispersity index (PDI), initial zeta potential (IZP), and final zeta potential (FZP). SD: standard deviation; * After 120 days of storage at 25 °C ± 2; ** After 190 days of storage at 25 °C ± 2. Filtration was performed using a 0.45 µm regenerated cellulose (RC) membrane filter.

**Table 3:** Larvicidal activity of *A. canelilla* essential oil (EOANIB), 1-nitro-2-feniletano (NFTANE), 1-Nitro-2-phenylethene (NFTENE) and formulations against *Aedes aegypti* larvae after 24 and 48 h.

Sample	Exposition Time	LC_50_ ± SEM (LCL-UCL) ppm	Slope ± SEM	LC_99_ ± SEM (LCL-UCL) ppm	Slope ± SEM	χ^2^ ppm
EOANIB	24 h	86.9 ± 0.8 (78.2–94.7) *	14.8 ± 2.0	133.1 ± 4.0 (117.5–175.7)	19.7 ± 3.4	62.5
	48 h	84.3 ± 0.6 (77.2–88.7)	22.9 ± 3.0	108.2 ± 2.3 (100.1–133.4)	29.6 ± 5.4	76.9
NFTANE	24 h	84.8 ± 0.5 (75.6–92.4) *	18.2 ± 2.0	131.4 ± 2.7 (116.1–172.5)	22.2 ± 3.1	62.8
	48 h	83.6 ± 0.6 (78.3–87.1) *	31.0 ± 3.0	100.1 ± 1.9 (94.6–114.9)	36.9 ± 8.0	85.4
NFTENE	24 h	10.9 ± 0.2 (8.0–14.0)	2.5 ± 0.1	24.8 ± 6.7 (19.9–35.7)	2.4 ± 0.1	66.8
	48 h	7.7 ± 0.1 (5.2–10.3)	3.7 ± 0.1	16.0 ± 1.2 (12.5–26.7)	3.7 ± 0.1	54.8
PEOANIB	24 h	67.6 ± 1.1 (59.4–76.0)	7.6 ± 0.9	106.3 ± 9.7 (92.5–140.9)	8.3 ± 1.3	66.5
	48 h	50.5 ± 0.8 (42.0–60.7)	6.8 ± 0.5	79.1 ± 7.0 (66.9–110.2)	5.8 ± 0.5	59.2
PNFTANE	24 h	63.9 ± 0.6 (57.9–70.6)	17.9 ± 2.0	93.9 ± 1.9 (83.1–119.3)	23.4 ± 3.0	96.4
	48 h	47.2 ± 0.6 (41.7–55.2)	6.7 ± 0.6	69.6 ± 7.5 (59.6–101.9)	6.2 ± 0.7	85.3
PNFTENE	24 h	6.5 ± 0.1 (4.9–8.3)	3.8 ± 0.3	13.6 ± 2.4 (10.8–21.2)	2.4 ± 0.2	51.3
	48 h	4.4 ± 0.1 (3.1–5.7)	5.2 ± 0.8	7.8 ± 2.0 (6.3–14.0)	3.7 ± 0.1	36.6

Positive control: Temephos, 1.0 ppm, 100% mortality; negative control: DMSO and NBr 0% mortality at 1% *v*/*v*; LC_50_, lethal concentration for 50% of larvae; LC_99_, lethal concentration for 99% of larvae; SD, standard deviation (Quadruplicates); LCL, lower confidence limit (95%); UCL, upper confidence limit (95%); *Aniba canelilla* essential oil (EOANIB); 1-Nitro-2-phenylethane (NFTANE); 1-Nitro-2-phenylethene (NFTENE); Chi-squared value (χ^2^). Note: LC (Lethal Concentration) values were calculated using nonlinear regression probit in STATGRAPHICS Centurion XV, Version 15.1.02. According to ANOVA performed in GraphPad Prism 9.0.2, *, the samples showed no statistically significant differences (*p* > 0.05). All other samples presented significant differences (*p* < 0.05).

**Table 4:** Adulticidal activity of essential oil of *A. canelilla* (EOANIB) against *Aedes aegypti* females.

Time (min)	LC_50_ ppm (LCL-UCL)	LC_90_ ppm (LCL-UCL)	LC_99_ ppm (LCL-UCL)	LC_99.9_ ppm (LCL-UCL)	χ^2^ ppm
15	75.2 (65.6–83.9)	119.2 (108.1–135.9)	155.0 (137.8–183.1)	181.1 (159.0–218.1)	144.3
30	52.7 (43.3–60.0)	80.8 (72.4–94.5)	103.7 (91.0–127.8)	120.4 (103.9–152.8)	149.9
45	43.5 (38.0–48.3)	74.8 (69.2–81.9)	100.4 (91.9–112.0)	119.0 (108.1–134.4)	390.5
60	38.7 (28.4–47.2)	70.6 (61.2–84.4)	96.5 (83.0–119.6)	115.5 (98.3–146.1)	127.1
75	38.0 (27.7–46.5)	69.9 (60.4–83.7)	95.8 (82.3–118.9)	114.9 (97.5–145.4)	126.2
90	33.9 (23.5–42.6)	65.5 (55.9–79.6)	91.3 (77.7–114.5)	110.2 (92.8–140.7)	122.3
1440	42.2 (32.0–50.5)	74.1 (64.9–87.9)	100.2 (86.7–123.4)	119.2 (101.9–150.1)	129.1

**Note:** LC (Lethal Concentration) 50, 90, 99 and 99.9 of EOANIB values were calculated using nonlinear regression Probit regression model in STATGRAPHICS Centurion XV, Version 15.1.02. LCL, lower confidence limit (95%); UCL, upper confidence limit (95%); Assay Quadruplicate, χ^2^: Chi-square value. According to ANOVA performed in GraphPad Prism 9.0.2, the samples EOANIB 45 min and EOANIB 24 h showed no statistically significant differences (*p* > 0.05); positive control: Deltamethrin K-Othrine^®^ at 10 ppm, 100% mortality; negative control: Acetone, 0% mortality. Post-transfer conditions: temperature 26 ± 1 °C, relative humidity 80 ± 5%.

**Table 5:** Toxicity test on *Artemia salina* nauplii with EOANIB, NFTANE, NFTENE, PEOANIB and PNFTANE.

Sample	LC_50_ ± SEM (CI) ppm	Slope ± SEM
EOANIB	* 77.2 ± 1.8 (73.8–81.2)	9.22 ± 1.9
NFTANE	* 98.15 ± 3.4 (75.6–92.4)	5.8 ± 0.9
NFTENE	1.9 ± 0.1 (1.8–2.1)	4.5 ± 0.6
PEOANIB	* 82.0 ± 1.7 (78.5–85.8)	9.15 ± 1.5
PNFTANE	* 91.2 ± 3.3 (82.2–98.7)	4.3 ± 0,7

**Note.** (CI): Lower and upper 95% confidence intervals, respectively. The samples * (EOANIB and NFTANE) have a statistical difference. The *p*-value was obtained by the Wilcoxon test for data that do not have normality and the *p*-value obtained was below <0.05, indicating statistical significance between the replicates. Negative control DMSO and NBr 0% mortality at 1% *v*/*v*.

**Table 6:** In vitro acetylcholinesterase enzyme (AChE) inhibitory activity of *Aniba canelilla* essential oil (EOANIB), 1-Nitro-2-phenylethane (NFTANE) and 1-Nitro-2-phenylethene (NFTENE); Means followed by different letters are significantly different (*p* < 0.05); IC50: inhibitory concentration 50% (µg∙mL^−1^), SEM: standard error of the mean (triplicates), 95% CI confidence interval of 95%, LCL: lower confidence limit, UCL: upper confidence limit, R^2^: R square value, galantamine: positive control.

Sample	IC_50_-SEM	CI 95% (LCL–UCL)	Slope ± SEM	R^2^
EOANIB	163.2 ± 7.03 ^a^	149.2–178.1	1.214 ± 0.062	0.969
NFTANE	189.7 ± 9.30 ^b^	171.6–209.4	1.175 ± 0.066	0.9579
NFTENE	148.9 ± 8.56 ^a^	131.7–167.4	1.283 ± 0.094	0.9401
Galantamine	0.72 ± 0.03	0.66–0.78	1.053 ± 0.053	0.9870

**Table 7:** In vitro inhibition of acetylcholinesterase enzyme from insect homogenates using galantamine and NFTENE as inhibitors. 1-Nitro-2-phenylethene (NFTENE); Means followed by different letters are significantly different (*p* < 0.05); IC_50_: inhibitory concentration 50% (µg∙mL^−1^), SD: standard deviation (triplicates), 95% CI: confidence interval of 95%, LCL: lower confidence limit, UCL: upper confidence limit, R^2^: R square value. Obs: * Gal: positive control galantamine at 100 µg∙mL^−1^.

Homogenate	Compound	Inhibition Rate (%) ± SD at 1.5 mg∙mL^−1^	IC_50_-SEM	CI 95% (LCL–UCL)	Slope ± SEM	R^2^
*A. aegypti* adults	NFTENE	34.0 ± 2.18	3264 ± 370.9	2630 ± 4354	0.86 ± 0.07	0.95
	Galantamine	95.01 ± 1.91 *	0.36 ± 0.06	0.22–0.48	0.68 ± 0.08	0.97
*A. aegypti* larvae	NFTENE	71.8 ± 2.21	672.3 ± 67.45	551.5–837.8	0.72 ± 0.06	0.92
	Galantamine	98.3 ± 0.05 *	0.30 ± 0.04	0.21–0.40	0.57 ± 0.05	0.97
*D. melanogaster* adults	NFTENE	18.4 ± 2.11	3135 ± 281.31	2615–4039	2.02 ± 0.20	0.97
	Galantamine	99.8 ± 0.41 *	1.2 ± 0.17	0.87–1.59	0.73 ± 0.09	0.93

**Table 8:** Masses weighed in the preparation of nanomicelle formulations loaded with a sample of essential oil from *A. canelilla* leaves (EOANIB), isolated substance 1-Nitro-2-phenylethane (NFTANE) and synthetic derivative 1-Nitro-2-phenylethene (NFTENE).

Sample	SM (mg)	PM (mg)	[C] mg∙mL^−1^
PEOANIB	59.0	202.7	14.75
PNFTANE	57.7	201.8	14.43
PNFTENE	13.5	200.5	3.37
PBlank	0	200.3	0

SM: sample mass (mg); PM: pluronic mass (mg); volume of DMSO (100 µL); volume of water (4 mL).

## Data Availability

The original contributions presented in this study are included in the article/Supplementary Material. Further inquiries can be directed to the corresponding authors.

## Supplementary Materials

The following supporting information can be downloaded at: https://www.mdpi.com/article/10.3390/plants14213348/s1, Figure S1: Initial zeta potential (IPZ) distribution of PEOANIB, PNFTANE and PNFTENE (0 day); Figure S2: Final zeta potential (FZP) distribution of PEOANIB and PNTFANE (120 days) and PNFTENE (190 days); Table S1: Temperature-accelerated stability of PEOANIB, PNFTANE and NFTENE formulations. Micellar size, polydispersity index and transmittance; Table S2: Mean micellar size (nm) of PEOANIB, PNFTANE and PNFTENE and PdI after 190 days of storage at 25 °C.

## Abbreviations

The following abbreviations are used in this manuscript:

EOANIB	Essential oil leaves of *Aniba canelilla*
NFTANE	1-Nitro-2-phenylethane
NFTENE	1-Nitro-2-phenylethene
PEOANIB	Formulation Essential oil leaves of *Aniba canelilla*
PNFTANE	Formulation 1-Nitro-2-phenylethane
PNFTENE	Formulation 1-Nitro-2-phenylethene
AChE	Acetylcholinesterase
NTDs	Neglected tropical diseases
WHO	World Health Organization
DMSO	Dimethyl sulfoxide
BCA	Bicinchoninic acid
