# Supplementary material for: Insecticidal Potential of Aniba canelilla (H.B.K.) Mez Essential Oil Against Aedes aegypti: Larvicidal and Adulticidal Activities, Mechanism of Action, and Formulation Development
Source: Plants (Basel). 2025 Oct 31;14(21):3348. doi: 10.3390/plants14213348 (PMC12608320; doi:10.3390/plants14213348)

Insecticidal Potential of *Aniba canelilla* (H.B.K.) Mez Essential Oil Against *Aedes aegypti*: Larvicidal and Adulticidal Activities, Mechanism of Action, and Formulation Development

Jefferson D. da Cruz [1,2], Maíra M. H. Almeida [1,2], Maria Athana M. Silva [1], Jefferson R. A. Silva [3], Fernando A. Genta [4,*] and Ana Claudia F. Amaral [1,2,*]

[1] Laboratório de Produtos Naturais e Derivados, Departamento de Produtos Naturais, Farmanguinhos/Fiocruz, Manguinhos, Rio de Janeiro 21041-250, RJ, Brazil; jefferson_dacruz@hotmail.com (J.D.d.C.); maira-haddad@hotmail.com (M.M.H.A.); maria.mpalantinos@fiocruz.br (M.A.M.S.)
[2] Programa de Pós-Graduação Acadêmica em Pesquisa Translacional em Fármacos e Medicamentos, Farmanguinhos/Fiocruz, Manguinhos, Rio de Janeiro 21041-250, RJ, Brazil
[3] Laboratório de Cromatografia, Departamento de Química, Instituto de Ciências Exatas, Universidade Federal do Amazonas, Manaus 69077-000, AM, Brazil; jrocha_01@yahoo.com.br
[4] Laboratório de Bioquímica e Fisiologia de Insetos, Instituto Nacional de Endemias Rurais (INERu), IOC/Fiocruz, Rio de Janeiro 21040-360, RJ, Brazil
[5] Laboratório de Bioquímica e Fisiologia de Insetos, Instituto Oswaldo Cruz, IOC/Fiocruz, Rio de Janeiro 21040-360, RJ, Brazil
* Correspondence: genta@ioc.fiocruz.br (F.A.G.); aamaral_99@yahoo.com.br (A.C.F.A.)

*Corresponding author.

e-mail address: aamaral_99@yahoo.com.br (A.C.F. Amaral)

e-mail address: genta@ioc.fiocruz.br (F.A. Genta)

Table S1. Temperature-accelerated stability of PEOANIB, PNFTANE and NFTENE formulations. Micellar size, polydispersity index and transmittance.

**Temp °C**	**Hydrodynamic diameter (nm)**			**Particle size 1 ± SD. nm (Area. %)**			**Particle size 2 ± SD. nm (Area. %)**	**PDI**			**Transmittance %**		
	**PEOANIB**	**PENTANE**	**PNFTNE**	**PEOANIB**	**PENTANE**	**PNFTENE**	**PNFTENE**	**PEOANIB**	**PNFTANE**	**PNFTENE**	**PEOANIB**	**PNFTANE**	**PNFTENE**
**25**	33.4	32.4	370.3	28.7 ± 7.8a	38.4 ± 13.6a	399.2 ± 111.2 (95.1)	20.6 ± 2.6 (4.9)	0.194 ± 0.041	0.226 ± 0.033	0.206 ± 0.04	85.4	85.1	84.9
**30**	34.5	32.8	335.2	28.5 ± 5.8a	36.3 ± 14.0a	361.4 ± 126.9 (95.1)	15.9 ± 2.0 (4.9)	0.163 ± 0.056	0.198 ± 0.009	0.212 ± 0.056	85.6	85.6	84.9
**35**	32.1	32.4	310.1	34.2 ± 11.0a	33.3 ± 8.8a	380.2 ± 128.5 (88.6)	25.1 ± 3.6 (11.4)	0.142 ± 0.024	0.15 ±0.026	0.252 ± 0.029	85.6	85.7	84.7
**40**	32.9	32.1	250.3	24.5 ± 4.0a	33.2 ± 9.6a	354.3 ± 116.8 (84.4)	24.8 ± 3.9 (15.6)	0.121 ± 0.075	0.134 ± 0.045	0.238 ± 0.018	85.7	85.7	84.1
**45**	32.2	31.7	246.7	31.5 ± 6.9a	32.9 ± 8.8a	334.4 ± 107.5 (81.1)	25.7 ± 4.3 (18.9)	0.119 ± 0.05	0.117 ± 0.003	0.265 ± 0.02	85.3	85.7	84.5
**50**	31.7	31.3	347.0	30.5 ± 6.5a	32.7 ± 9.1a	383.2 ± 142.8 (81.5)	24.7 ± 3.8 (18.5)	0.088 ± 0.047	0.135 ± 0.024	0.267 ± 0.021	85.6	85.6	84.5
**55**	31.1	31	217.4	30.2 ± 6.6a	32.3 ± 8.9a	379.0 ± 154.8 (79.3)	26.3 ± 5.2 (20.7)	0.079 ± 0.045	0.099 ± 0.042	0.277 ± 0.036	85.6	85.7	84.7
**60**	30.8	30.6	291.7	30 ± 6.6a	31.0 ± 8.1a	335.3 ± 125.1 (78.7)	25.7 ± 4.8 (21.3)	0.064 ± 0.031	0.114 ± 0.038	0.263 ± 0.026	85.7	85.8	84.6
**25**	29.3	31.5	324,4	30.8 ± 0.2a	30.4 ± 0.42a	321.9 ± 40.2 (98.9)	16.0 ± 6.1 (1.1)	0.23 ± 0.008	0.233 ± 0.004	0.236 ± 0.031	85.3	85.1	76,4
**Average**	32.0	31.8	296.1	29.9	33.4	365	23.6	0.133	0.156	0.248	85.5	85.6	84.6
**St.Dev.**	1.5	0.7	54.2	2.6	2.5	23.5	3.6	0.055	0.05	0.035	0.2	0.3	0.26
**RSD%**	4.8	2.3	18.3	8.8	7.5	6.42	15.1	41.2	32.1	14.2	0.2	0.3	0.31

Note: All dilutions were made with 50uL/1mL of water. The proportion of peak 1 is relative to 100% of the distribution. S = Standard deviation. RSD% = Relative Standard Deviation (M/S)*100. NA = Not available. Area % = Area corresponding to the percentage of particles in the formulation when the formulation presents a bimodal distribution. a = Monomodal distribution.

Table S2. Mean micellar size (nm) of PEOANIB, PNFTANE and PNFTENE and PdI after 190 days of storage at 25°C.

Time (days)	Hydrodynamic diameter (nm)			Particle size 1 ± SD (nm)			PDI			Transmittance %		
	PEOANIB	PNFTANE	PNFTENE	PEOANIB	PNFTANE	PNFTENE	PEOANIB	PNFTANE	PNFTENE	PEOANIB	PNFTANE	PNFTENE
**0**	33 ± 0.6	32.1 ± 0.3	370.0	35.2 ± 5.6	33.6 ± 4.3	329.6 ± 63.0	0.195 ± 0.004	0.225 ± 0.004	0.129 ± 0.017	85.3 ± 0.1	85.3 ± 0.2	83.4
**7**	39.5 ± 17.1	29.8 ± 0.9	NA	32.5 ± 4.3	34.9 ± 3.3	NA	0.213 ± 0.019	0.223 ± 0.015	NA	85.9 ± 0.7	86.0 ± 0.1	NA
**10**	NA	NA	373.5	NA	NA	342.2 ± 76.9	NA	NA	0.256 ± 0.034	NA	NA	77.1
**15**	30.6 ± 3.5	30.1 ± 1.0	NA	31.8 ± 1.9	31.9 ± 2.2	NA	0.202 ± 0.036	0.219 ± 0.016	NA	87.2 ± 0.2	86.8 ± 0.1	NA
**30**	28.4 ± 0.9	33.5 ± 2.4	NA	30.8 ± 2.8	31.5 ± 1.8	NA	0.192 ± 0.048	0.196 ± 0.041	NA	86.9 ± 0.3	86.5 ± 0.1	NA
**45**	26.7 ± 0.4	29.6 ± 1.4	NA	28.3 ± 0.8	30.3 ± 1.7	NA	0.15 ± 0.023	0.194 ± 0.048	NA	87.6 ± 0.4	87.2 ± 0.1	NA
**60**	28.9 ± 0.2	30.7 ± 1.2	NA	28.6 ± 0.1	31.3 ± 1.0	NA	0.073 ± 0.023	0.174 ± 0.039	NA	86.3 ± 0.1	85.3 ± 0.1	NA
**90**	NA	NA	376.9	NA	NA	397.7 ± 157.6	NA	NA	0.243 ± 0.035	NA	NA	74.6
**120**	27.2 ± 0.7	28.2 ± 1.1	NA	27.9 ± 1.2	30.3 ± 2.4	NA	0.125 ± 0.032	0.159 ± 0.043	NA	86.7 ± 0.2	85.2 ± 0.6	NA
**150**	NA	NA	399.1	NA	NA	414.0 ± 165.9	NA	NA	0.238 ± 0.022	NA	NA	69.1
**190**	NA	NA	354.1	NA	NA	386.4 ± 68.0	NA	NA	0.177 ± 0.022	NA	NA	72.8
**Average**	30.6	28.8	374.7	30.7	32	374.0	0.164	0.198	0.209	86	86	75.4
**St.Dev.**	4.4	5.8	16.2	2.7	1.3	36.4	0.051	0.025	0.054	1.2	0.8	5.3
**RSD%**	14.6	20.3	4.3	8.7	4.1	9.7	31	12.8	25.8	1.4	0.9	7.1

Note: All dilutions were prepared using 50 µL in 1 mL of water. The proportion of peak 1 corresponds to 100% of the distribution. S = standard deviation. RSD% = relative standard deviation, calculated as (mean/S) × 100.

Figure S1. Initial zeta potential (IPZ) distribution of PEOANIB, PNFTANE and PNFTENE (0 day). Temperature 25°C, Equilibration time 1 min, Henry factor 1.5 (Smoluchowski); Adjusted voltage 200 V, solvent water, Solvent refractive index: 1,3303, Solvent viscosity: 0,0008903 Pa.s, solvent relative permitivity: 78.37, Mean zeta potential. Distribution peak line (dotted line), Conductivity: 0.01 mS/cm, Dilution: 1:20.

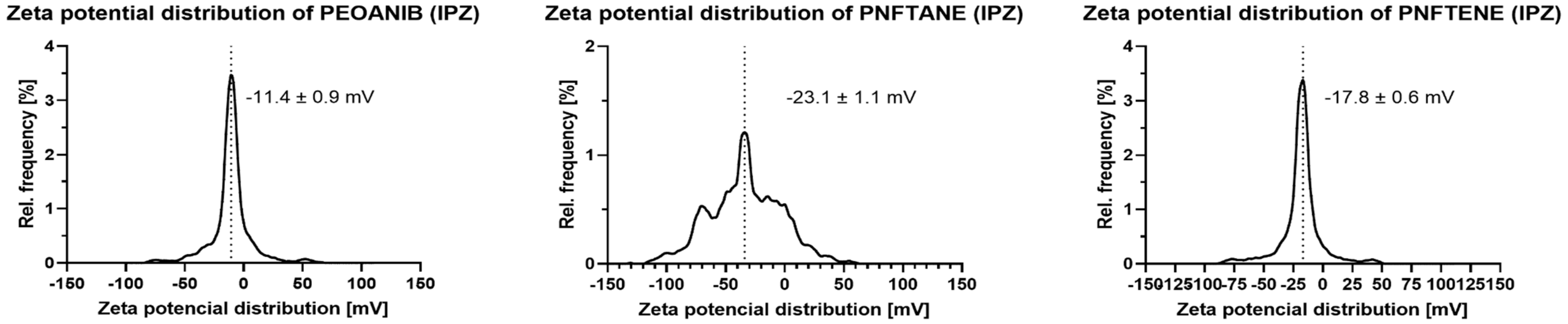

Figure S2. Final zeta potential (FZP) distribution of PEOANIB and PNTFANE (120 days) and PNFTENE (190 days). Temperature 25°C, Equilibration time 1 min, Henry factor 1.5 (Smoluchowski); Adjusted voltage 200 V, solvent water, Solvent refractive index: 1,3303, Solvent viscosity: 0,0008903 Pa.s, solvent relative permittivity: 78.37, Mean zeta potential, Distribution peak line (dotted line), Conductivity: 0.01 mS/cm, Dilution: 1:20.

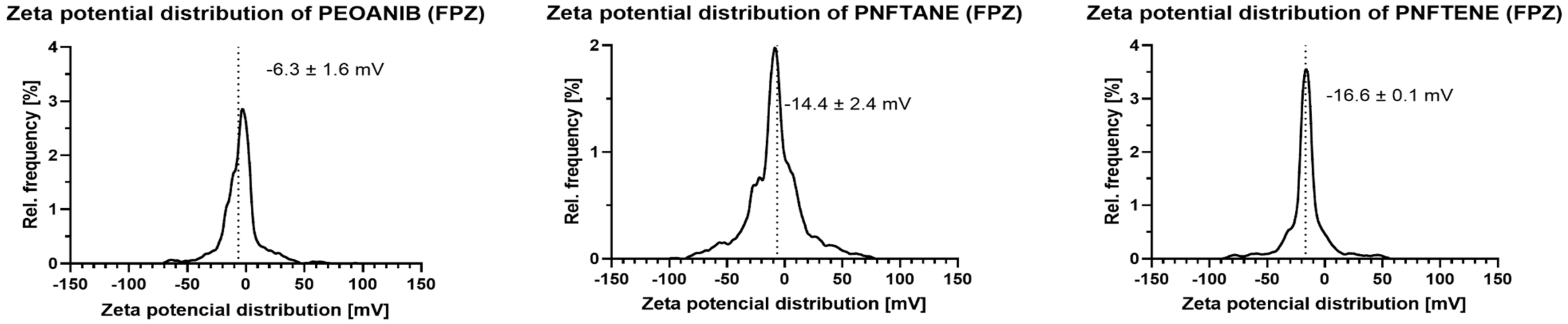

Supplement: Supplementary file 1 [file plants-14-03348-s001.zip › plants-3926380-supplementary.pdf]
